# Characterization of complete chloroplast genome and phylogenetic analysis of sweet cherry *Cerasus avium* (L.) Moench (Prunoideae, Rosaceae)

**DOI:** 10.1080/23802359.2018.1532835

**Published:** 2018-10-31

**Authors:** Tao Chen, Guo-Ping Hu, Yan Wang, Qing Chen, Lei Wang, Jing Zhang, Hao-Ru Tang, Xiao-Rong Wang

**Affiliations:** a College of Horticulture, Sichuan Agricultural University, Chengdu, China;; b Institute of Pomology and Olericulture Sichuan Agricultural University, Chengdu, China

**Keywords:** *Cerasus avium*, chloroplast genome, Illumina sequencing, phylogenetic analysis

## Abstract

Sweet cherry (*Cerasus avium* (L.) Moench) belonging to family Rosaceae, is an important economical fruit crop worldwide. In this study, the complete chloroplast (cp) genome of sweet cherry was generated by *De novo* assembly with low coverage whole-genome sequencing data. The genome size was 157,987 bp in length consisting of a typical quadripartite structure; a large single-copy region (LSC, 85,975 bp), a small single-copy region (SSC, 19,121 bp) and a pair of inverted repeat regions (IRs, 26,445 bp each). A total of 115 genes were predicted including 82 protein-coding genes, 29 tRNA genes and four rRNA genes. Phylogenetic analysis based on 12 reported complete chloroplast genome indicated the monophyly of the genus *Creasus* including newly sequenced *C. avium*, which is conform to the traditional classification.

Sweet cherry (*Cerasus avium* (L.) Moench) is one of the four cultivated cherry species worldwide. Its production has increased over 30% during the last two decades (Calle et al. 2018). It is reported that cultivated sweet cherry was domesticated from its wild ancestor ‘Mazzard’ in 4000–5000 years ago and also revealed one domestication event (Janick 2005; Meyer and Purugganan 2013). Despite the wide distribution and ample species of genus *Cerasus*, the genetic relationship of *C. avium* relative to other cherries has not been well established in the previous phylogenetic analyses using several nuclear and chloroplast markers (Pervaiz et al. 2015), especially for Chinese cherry (*Cerasus pseudocerasus*) and sweet cherry (*C. avium*) (Shi et al. 2013). Here, we generated the complete chloroplast genome sequence of *C. avium* to elucidate the phylogenetic relationship between *C. avium* and other cherries.

Total genomic DNA of ‘Mazzard’ was isolated from fresh leaves sampled from national fruit germplasm repository (kindly provided by Zhengzhou Fruit Research Institute, Chinese Academy of Agricultural Sciences) using modified CTAB protocol. Voucher specimen .was deposited in Sichuan Agricultural University. Illumina paired-end (PE) library with 500-bp insert size was constructed and sequenced using Illumina HiSeq 2500 platform (Illumina, San Diego, CA). After quality trimming, a total of 4.23 Gb clean PE reads (Phred scores >20) were assembled using SOAPdenovo software (Li et al. 2009). Contigs were ordered and merged with the chloroplast sequence of *Cerasus persica* (NC_04697). Genome annotation was performed with Dual Organellar Geno Me Annotator (DOGMA) (Wyman et al. 2004) (http://dogma.ccbb.utexas.edu/)

The circular DNA of *C. avium* was 157,987 bp in length consisting of four regions; large single copy region (LSC) of 85,975 bp, small single copy region (SSC) of 19,121 bp, and a pair of inverted repeat regions (IRs) of each 26,445 bp. The overall GC contents were 35.72%. A total 115 unique coding regions were predicted, comprising 82 protein-coding genes, 29 tRNA genes, and four rRNA genes. Among all unique genes, nine genes contain one intron, two genes (*ycf3* and *clpP*) with two introns. All the coding regions accounted for 57.36% of the whole genome. The genome was deposited in GenBank (MH_756631).

Similarity complete chloroplast genome sequence of other 12 Rosales species (*Pyrus pyrifolia* NC_015996 and *Pyrus spinosa* NC_023130 as outgroups) were aligned using MAFFT 5 (Katoh et al. 2005). Maximum-likelihood (ML) analysis was implemented in RAxML v8.2.4 (Stamatakis 2006). Maximum parsimony (MP) and neighbour-joining (NJ) analysis were performed using MEGA 6.0 (Tamura et al. 2013) (http://www.megasoftware.net/).

**Figure 1.**
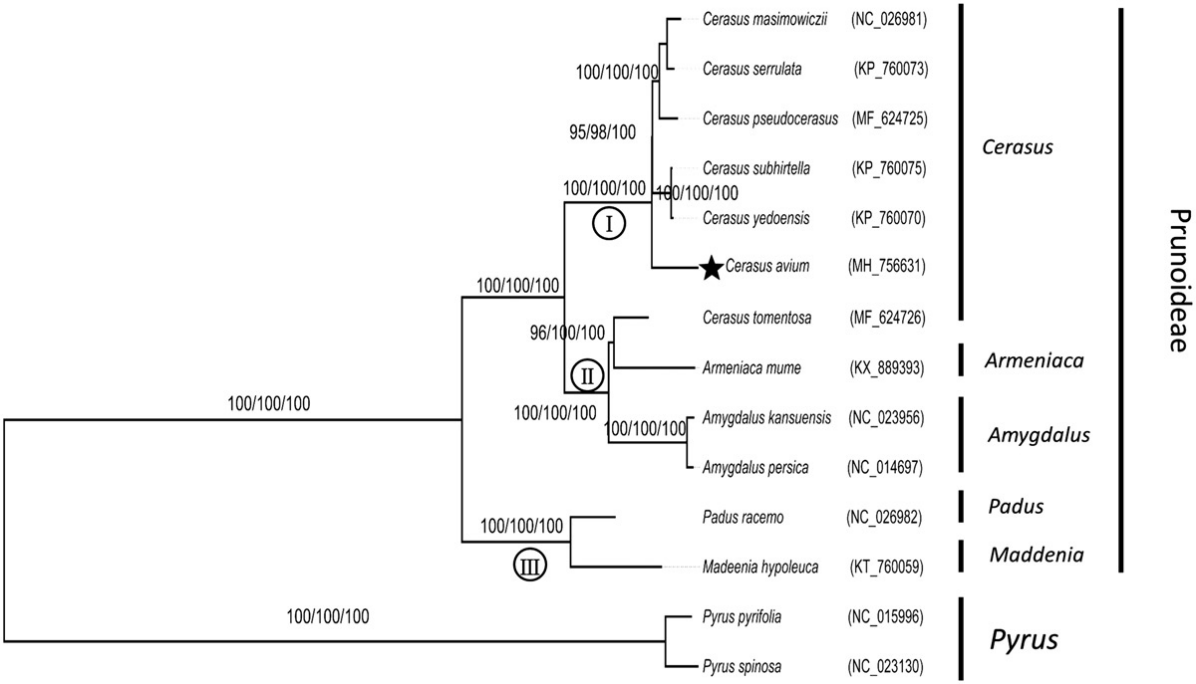
Phylogenetic tree of *Cerasus avium* with other 13 species belonging to Prunoideae, Rosaceae family. Tree was inferred from the complete chloroplast genome sequences using the ML method with a GTR model, MP method, and NJ method with a K-2P model. Only the framework of the ML tree was presented. Numbers in the nodes were the bootstrap values from 1000 replicates with an arrangement of ML/MP/NJ methods. Symbol (I, II, III) in the nodes represent three clades in subfamily Prunoideae.

Phylogenetic analysis revealed three clades in subfamily Prunoideae (Figure 1). *C. avium* was nested within genus *Cerasus*, which was a sister to other five *Creasus* species. *C. tomentosa* was nested within genus *Armeniaca*, and a sister to genus *Amygdalus* formed another clade. Genus *Padus* and *Maddenia* composed the third clade. This result was congruent with previous studies by other molecular markers (Wen et al. 2008; Chin et al. 2010; Chen et al. 2018).
